# Construction of a Porcine Reproductive and Respiratory Syndrome Virus with Nanoluc Luciferase Reporter: a Stable and Highly Efficient Tool for Viral Quantification Both *In Vitro* and *In Vivo*

**DOI:** 10.1128/spectrum.00276-22

**Published:** 2022-06-27

**Authors:** Yang Wang, Xinna Ge, Yongning Zhang, Xin Guo, Jun Han, Lei Zhou, Hanchun Yang

**Affiliations:** a Key Laboratory of Animal Epidemiology of Ministry of Agriculture and Rural Affairs, College of Veterinary Medicine, China Agricultural University, Beijing, People’s Republic of China; University of Georgia

**Keywords:** porcine reproductive and respiratory syndrome virus (PRRSV), NanoLuc luciferase reporter, viral quantification, high-throughput screening, antiviral reagents, virus neutralization assay, pathogenicity

## Abstract

Porcine reproductive and respiratory syndrome virus (PRRSV) is one of the most economically important pathogens for the global pork industry, characterized for its genetic variation and unsatisfied heterological protection from vaccines. A high-throughput screening platform for developing anti-PRRSV therapies is urgently needed. Here, an 11-amino-acid subunit HiBiT derived from NanoLuc luciferase was inserted into the PRRSV genome at four loci of the Nsp2 coding region or as an additional TRS2 driven open reading frame (ORF) between the ORF7 and 3′-untranscribed region (3′-UTR), respectively, and five recombinant viruses with luciferase activity were successfully rescued. The virological characteristics of the representative virus RvJX-Nsp2_325_-HiBiT were investigated. *In vitro*, it displayed similar growth kinetics as the parental virus and keeps the luciferase activity and genetic stability after eight rounds of serial passages. The concept-proof test confirmed that RvJX-Nsp2_325_-HiBiT can be easily used to evaluate the efficacy of antiviral reagents by detecting the reduction of luciferase activity, showing a consistent trend with infectious titers, as well as to set a novel convenient virus neutralization assay based on the intensity of luciferase activity. Last, the viral proliferation, virulence, validity, and HiBiT stability were further investigated in pig inoculation study, showing that the luciferase activity can be directly detected in the tissue samples or indirectly from the MARC-145 cells inoculated with sera from RvJX-Nsp2_325_-HiBiT-inoculated pigs. Taken together, the results indicate that the HiBiT-tagged virus is a convenient and stable tool for evaluating viral propagation both *in vitro* and *in vivo*, which can provide a high-efficient platform for screening and evaluating anti-PRRSV therapies.

**IMPORTANCE** Luciferase reporter tagged virus is crucial to viral quantification in the study of viral replication, pathogenesis and exploring antiviral reagents. It is urgently needed for PRRSV academia to construct a stable, fast, and high-throughput reporting system, which can be used both *in vitro* and *in vivo.* Here, an 11-amino-acid luciferase subunit was successfully inserted into the PRRSV genome; the feasibility, genetic stability, and efficiency for viral quantification both *in vitro* and *in vivo* were characterized; and the results demonstrated it has greatly improved the convenience and efficiency for screening the anti-PRRSV reagents. Furthermore, a novel luciferase-based virus neutralization assay was successfully set, which can eliminate the step of sample gradient dilution and greatly improve the convenience and throughput of neutralizing antibody testing. Predictably, it will greatly facilitate the screening and evaluating anti-PRRSV therapies, as well as the mechanistic study of its replication and pathogenesis in the future.

## INTRODUCTION

Porcine reproductive and respiratory syndrome (PRRS), characterized by reproductive failure in breeding sows and respiratory distress in pigs of all ages, initially emerged in North America and Western Europe in the late 1980s (1, 2). In the last 30 years, this economically important disease has become endemic in most pig-producing countries worldwide (3). The causative agent, PRRS virus (PRRSV), is an enveloped, positive-stranded RNA virus, belonging to the genus Porartervirus of the family *Arteriviridae* within the order *Nidovirales* (4) Based on the difference of the genome, PRRSV can be grouped into two genotypes: the European genotype (type I) and the North American genotype (type II), represented by prototype viruses Lelystad and VR-2332, respectively (5, 6). As an RNA virus with low replication fidelity, PRRSV is known for its genomic and antigenic diversity and strain-specific variation in the field, which can seriously reduce the efficiency of heterological cross-protection from vaccines (7). Since many effective virostatics targeting various viral replication processes have been developed to treat important viral pathogens, such as hepatitis C virus and human immunodeficiency virus (8), especially during the pandemic of severe acute respiratory syndrome coronavirus 2 (SARS-CoV-2), many antiviral strategies are studied to develop the effective treatment for coronavirus disease 2019 (COVID-19) (9). Currently, veterinarians and researchers have a strong inclination to discover effective reagents to control this economically important disease. Therefore, high-throughput and easy-to-perform platforms for antiviral reagent screening are urgently needed.

The PRRSV genome is approximately 15 kb in length and contains at least 12 identified open reading frames (ORFs) with the 5′-cap and 3′-polyadenylated tail (10, 11). Two overlapping ORF1a and ORF1b covered three-quarters of the genome, encoding the two viral replicase proteins pp1a and pp1ab, which are further processed into 16 nonstructural proteins (Nsps), which are responsible for viral RNA synthesis during transcription and replication (12). Among the nonstructural protein coding regions, the Nsp2 is one of the most variated regions with genetic variation, deletion, and even insertion. The rest of the genome at the 3′ terminus encodes the structural proteins, including E, GP2, GP3, GP4, GP5, GP5a, M, and N proteins, in the order 5′-ORF1-E-GP2-GP3-GP4-ORF5a-GP5-M-N-3′, which are expressed by a nested series of subgenomic (sg) RNAs, produced during viral transcription. The structure of sg mRNAs derives from the discontinuous step of minus-strand RNA synthesis, which is guided by conserved AU-rich transcription-regulating sequences (TRS) (13).

Benefiting from the development of the viral reverse genetics system, recombinant or modified viruses can be generated to characterize virus biology in different aspects. Especially the recombinant viruses possessing reporter proteins have been widely used to elucidate the virus life cycle, to discover the manner of spreading *in vivo*, and to screen the antiviral reagents (14, 15). The reporter proteins, including naturally occurring fluorescent proteins and genetically engineered derivatives, are usually inserted into the viral genome, and then the bioimaging of viral infection is achieved by the expression of reporter proteins, which is an effective tool to detect and quantify viral replication *in vitro* and *in vivo* (16–18). For PRRSV, both strategies fusion expressing and setting as an additional ORF have been utilized to express the foreign green fluorescent protein (GFP) and/or red fluorescence protein (RFP) (19, 20). Considering the Nsp2 coding region could tolerate insertion and deletion, the GFP was first inserted into a unique deletion site located at amino acid positions 348 and 349 of the Nsp2 region in a genotype I PRRSV (21). Later, an enhanced GFP (EGFP) gene linked with TRS at its 5′ terminus was also inserted between ORF1b and ORF2a or between ORF7 and 3′-UTR, respectively, which confirmed that the TRS of PRRSV can effectively modulate the transcription and expression of foreign EGFP as a separate ORF (22, 23). Initially, the reporter genes were not stable in the PRRSV genome, as the recombinant PRRSV with GFP fused in the Nsp2 region lost its GFP-expressing after passaging in cells seven times, due to partial deletion of the GFP gene (21). Recently, the coexpression of RFP and GFP in PRRSV genome has been reported to be genetically stable during 20 serial passages in MARC-145 (20). Although the stability of the foreign EGFP gene increased, as an individual ORF, the recombinant viruses cannot be rescued when a larger reporter proteins firefly luciferase (FLuc) was inserted at the same position (24). Even though the fluorescence proteins such as EGFP, GFP, and RFP are good markers for visualizing the virus infection, they are still inconvenient for qualifying the viral replication and proliferation; therefore, nowadays, it is still time-consuming to titer the PRRSV or evaluate its replication efficiency.

To figure out the inconvenience of lacking a fast and high-throughput reporting system, here NanoLuc binary technology (NanoBiT) and reverse genetic operation were employed to rescue several recombinant viruses with inserted NanoBiT fragments (25, 26). NanoBiT is a split reporter, consisting of two subunits of different sizes, large NanoBiT (LgBiT, 158 amino acids [aa]) and high-affinity NanoBiT (HiBiT, 11 aa). Compared with the GFP/RFP applied in previous study, the HiBiT tag has nonnegligible advantages, such as its small size, benefiting the operation of recombinants and the stability of tag. In addition, it is much easier to quantify through luminescence by detecting the luciferase activity by “add-read” method. Last, bioluminescence of Nanoluc has much better sensitivity and dynamic range, shown as a higher signal:noise ratio.

In this study, the fragment of the HiBiT subunit was inserted into the PRRSV genome at different loci with two expression strategies to generate luciferase-tagged recombinant viruses. The results showed that the rescued recombinant virus displayed similar growth kinetics as the parental virus, and the infectious titers and luciferase of the recombinant viruses were stable after eight rounds of serial passages in cells. The convenience of this reporting system for screening antiviral reagents was also confirmed by testing the antiviral effect of four nucleoside analogs, because the decrease of luciferase activity was correlated with reduced infectious titers of recombinant virus, and a novel luciferase-based PRRSV neutralization assay was also developed, with higher throughput and a more convenient process compared with the conventional one. Furthermore, an animal inoculation experiment was also set to indicate that the recombinant virus was stable *in vivo* and can be used to monitor viral dynamics in serum and tissue. Collectively, our findings revealed that the split-luciferase reporter system is a practically helpful tool for evaluating viral propagation both *in vitro* and *in vivo* that can provide a highly efficient platform for screening and evaluating anti-PRRSV therapies.

## RESULTS

### Construction and recovery of HiBiT-tagged PRRSV.

**FIG 1:**
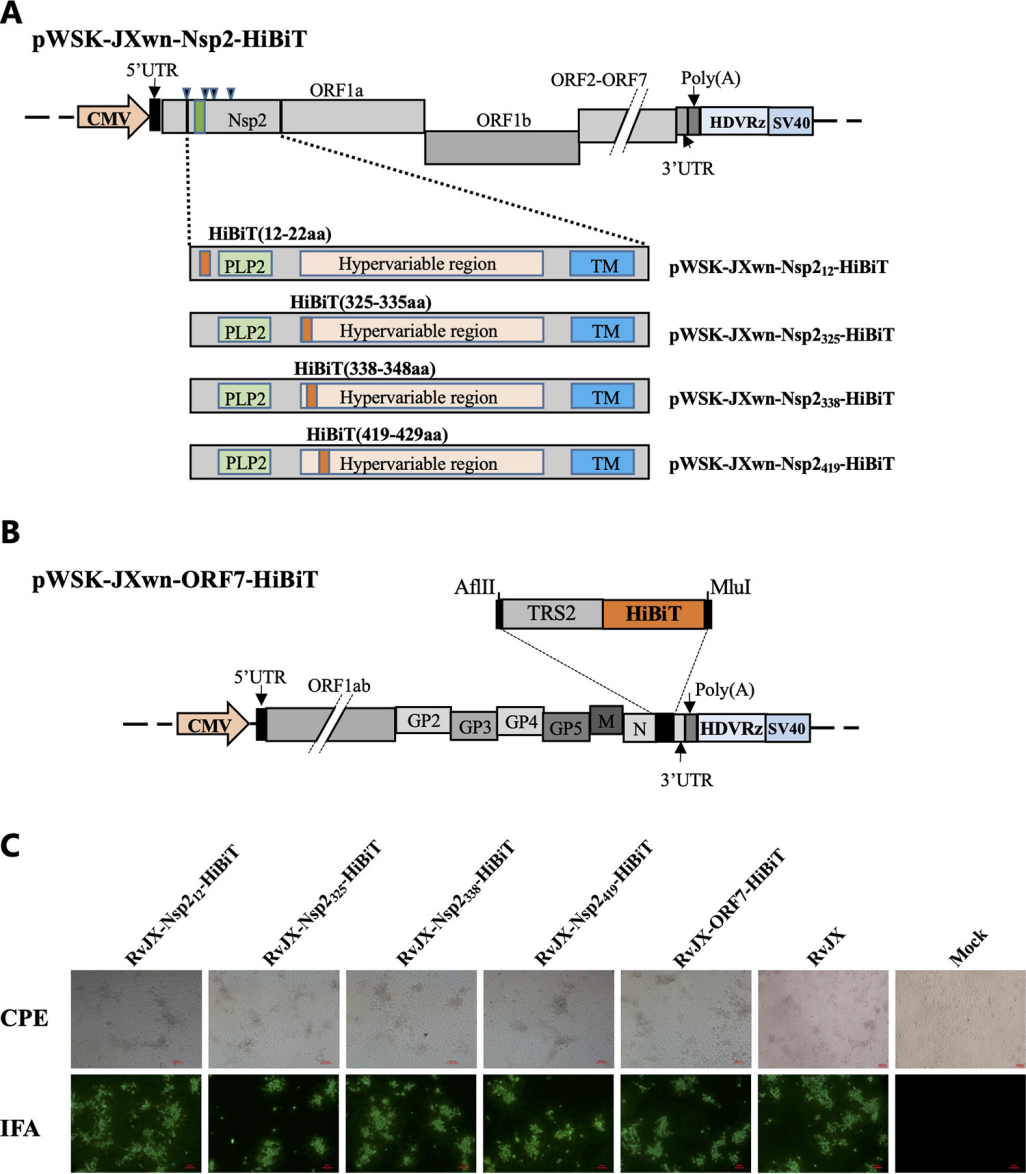
Construction and rescue of the recombinant porcine reproductive and respiratory syndrome virus (PRRSV) carrying the HiBiT gene. (A) Schematic diagram of PRRSV full-length infectious clones with HiBiT gene inserted into Nsp2 coding region. (B) Schematic diagram of PRRSV full-length infectious clones with HiBiT gene inserted between open reading frame 7 (ORF7) and 3´-untranscribed region (3´-UTR). The HiBiT gene fused with PRRSV TRS2 was inserted at the C terminus of the ORF7 to create a new ORF. (C) The recombinant PRRSVs were successfully rescued and confirmed by cytopathic effect (CPE) and immunofluorescence assay (IFA) on the MARC-145 cells. CMV, cytomegalovirus; PLP2, papain-like protease 2; TM, transmembrane; TRS, transcription-regulating sequence.

The fragments of aa 12 to 35 and aa 324 to 434 in the PRRSV Nsp2 coding region have been confirmed to be dispensable for viral replication (27), so we accordingly replaced the fragments of Nsp2 aa 12 to 22, aa 325 to 335, aa 338 to 348, or aa 419 to 429 with HiBiT tag to generate four recombinant PRRSV infectious clones, which were designated pWSK-JXwn-Nsp2_12_-HiBiT, pWSK-JXwn-Nsp2_325_-HiBiT, pWSK-JXwn-Nsp2_338_-HiBiT, and pWSK-JXwn-Nsp2_419_-HiBiT, respectively. In another full-length plasmid, the HiBiT fragment was inserted into the region between the ORF7 and the 3′-UTR, combined with a transcription regulatory sequence (TRS) of PRRSV ORF2, named pWSK-JXwn-ORF7-HiBiT (Fig. 1B). The PRRSV full-length plasmids were transfected into MARC-145 cells. Subsequently, the virus-induced cytopathic effect (CPE) was observed, characterized by cellular rounding and clumping; in addition, the expression of the PRRSV N protein was also confirmed by immunofluorescence assay (IFA). These results indicate that the recombinant viruses had been successfully rescued (Fig. 1C). Furthermore, at the third passage, the HiBiT tags were confirmed to exist in the PRRSV genome by sequencing the replaced or inserted regions and their flanking areas of viruses, which were found consistent with the original sequences with no additional mutation introduced (data not shown). The five rescued recombinant viruses were individually named RvJX-Nsp2_12_-HiBiT, RvJX-Nsp2_325_-HiBiT, RvJX-Nsp2_338_-HiBiT, RvJX-Nsp2_419_-HiBiT, and RvJX-ORF7-HiBiT.

### Characterization of the recombinant PRRSVs carrying HiBiT luciferase tag.

**FIG 2:**
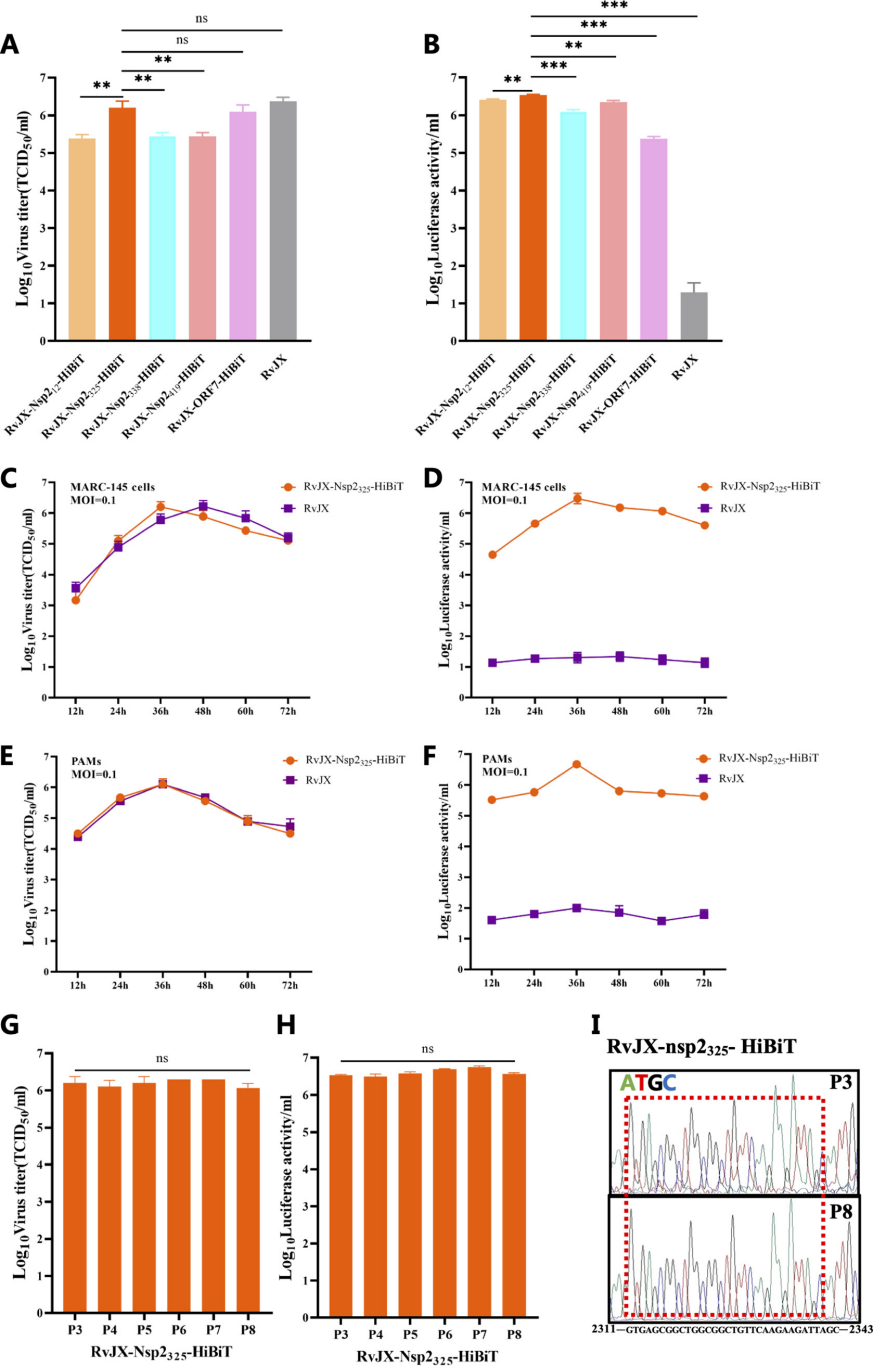
Characterization of the recombinant PRRSV *in vitro*. (A, B) Infectious titers and luciferase activity were determined upon infection with the recombinant PRRSV carrying the HiBiT gene at different loci. (C to F) *In vitro* growth kinetics of the recombinant PRRSV RvJX-Nsp2_325_-HiBiT on MARC-145 cells and pulmonary alveolar macrophages (PAMs), which were infected with parental RvJXwn and RvJX-Nsp2_325_-HiBiT at an multiplicity of infection (MOI) of 0.1. The virus titers and luciferase activity were determined at the indicated points, respectively. (G) Recombinant RvJX-Nsp2_325_-HiBiT was serially passaged on MARC-145 cells for eight rounds, and the virus titers of each passage were determined. (H) Luciferase activities of cells infected with the recombinant virus RvJX-Nsp2_325_-HiBiT at different passages. (I) Comparison of the sequences of the HiBiT gene in the recombinant virus RvJX-Nsp2_325_-HiBiT at the third and the eighth passages. Asterisks in panels A and B indicate significant differences between the RvJX-Nsp2_325_-HiBiT and other groups, on the average luciferase activity, or virus titer. *, *P < *0.05; **, *P < *0.01; ***, *P < *0.001. ns, no significant difference.

To determine the suitable locus for insertion of HiBiT into the PRRSV genome, the infectious titers and luciferase activities of all rescued viruses with HiBiT-tag were evaluated at the third passage. The titers of all recombinant virtues could reach 10^5^ to 10^6^ TCID_50_/mL, which were close to their parental backbone virus RvJXwn (abbreviated as RvJX in the figures). Among them, the recombinant virus RvJX-Nsp2_325_-HiBiT had the highest titer (Fig. 2A). Meanwhile, the luciferase activity of these recombinant viruses could reach log_10_ 5 to log_10_ 6/mL; in parallel, the luciferase activity of RvJXwn was only log_10_ 1/mL, which was regarded as the background value of this system (Fig. 2B). The results indicated that all five recombinant viruses were successfully rescued, in which the carried HiBiT tags can all correctly express and show luciferase activity when combined with LgBiT. In addition, the RvJX-Nsp2_325_-HiBiT shows the highest viral titer, as well as luciferase activity; therefore, it was selected as the represent virus for further characterization.

The growth kinetics of the recombinant virus RvJX-Nsp2_325_-HiBiT and its parental backbone virus RvJXwn were first compared on MARC-145 cells, and the recombinant virus can reach a titer that was comparable to that of the parental virus, but it reached the peak titer 12 h earlier (Fig. 2C). The growth kinetics of these two viruses were more consistent in pulmonary alveolar macrophages (PAMs), the target cells of PRRSV (Fig. 2E).

To investigate whether there is a correlation between infectious titers and luciferase expression, we simultaneously measured the luciferase activity of the samples used to determine the growth kinetics above. The data indicated that this recombinant virus RvJX-Nsp2_325_-HiBiT exhibited high infectivity on both MARC-145 cells and PAMs, and the trend of intracellular luciferase activity change was consistent with that of viral titer. To further examine the genetic stability of inserted HiBiT gene in the rescued virus, the virus was serially passaged in MARC-145 cells to the eighth passage, and then the infectious titers, luciferase activity, and sequence of the HiBiT gene were all checked. The data show that the luciferase activity and viral titers were well maintained during passaging, and no mutation was observed in the HiBiT tag and its flanking regions, suggesting that the recombinant PRRSV carrying split-luciferase (HiBiT) is genetically stable during eight serial passages in MARC-145 cells.

### Applicability of HiBiT-tagged PRRSV for antiviral reagents screening.

**FIG 3:**
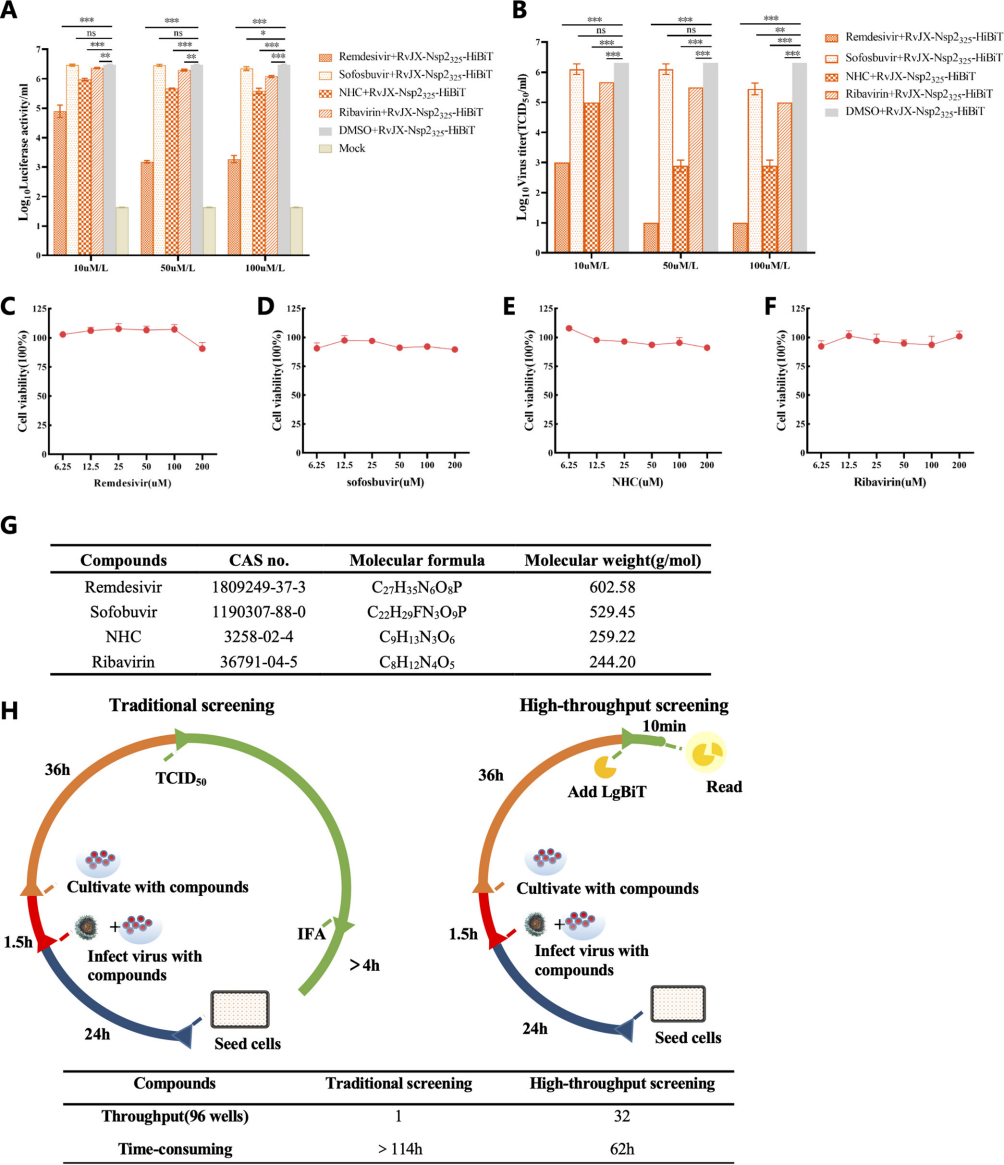
Application of the HiBiT-tagged PRRSV for antiviral reagents screening. (A, B) MARC-145 cells infected with the HiBiT-tagged PRRSV at MOI 0.1 were treated with remdesivir, sofosbuvir, β-d-*N*^4^-hydroxycytidine (NHC), and ribavirin at 1.5 h postinfection. Then infectious titers and luciferase activity levels were determined at 36 h postinfection (hpi), respectively. (C to F) Viability of cells treated with the four antiviral reagents was tested by using an 3-(4,5-dimethyl-2-thiazolyl)-2,5-diphenyl-2H-tetrazolium bromide (MTT) cell proliferation and cytotoxic assay kit and is shown as the percentage of viability, respectively. (G) Information for the four antiviral reagents used in this study. (H) Comparison of time-consuming and throughput between the traditional screening assay and the luciferase-based screening assay. Asterisks indicate a significant difference between the antiviral reagent and DMSO group, on the average luciferase activity, or virus titer. *, *P < *0.05; **, *P < *0.01; ***, *P < *0.001. DMSO, dimethyl sulfoxide.

As current commercial vaccines cannot provide satisfactory protection against the genetically diverse strains in the field, together with PRRSV modified live vaccines (MLVs) have the risk of reversion to virulence (7), the antiviral drug is becoming a tool pursued for PRRSV control. Therefore, an unbiased high-throughput screening system is urgently required. In this study, to determine the sensitivity of the HiBiT recombinant PRRSV to antiviral reagents, MARC-145 cells were first inoculated with RvJX-Nsp2_325_-HiBiT and treated with various concentrations of remdesivir, sofosbuvir, β-d-*N*^4^-hydroxycytidine (NHC), and ribavirin, respectively, which have been previously reported to show different levels of antiviral activity against other viruses (28–31). At 36 h postinfection, the infectious titer and luciferase activities of RvJX-Nsp2_325_-HiBiT were both determined. As shown in Fig. 3A, the luciferase activity of the recombinant virus was significantly reduced in remdesivir-, NHC-, and ribavirin-treated groups, but there was no obvious inhibition effect of sofosbuvir on PRRSV. The antiviral activity was further confirmed by the data of viral titer reduction. Additionally, the cell viability assays were carried out to ensure that tolerable concentrations of these drugs were all over 100 μM/liter on MARC-145 cells (Fig. 3C to F), which can exclude the possibility that the reduction of viral replication was due to the unspecific cytotoxicity of these reagents.

To compare the efficiency of the screening system based on luciferase activity or traditional viral titer testing by IFA, the throughput and time consumed for each process were recorded and calculated. As Fig. 3H shows, this HiBiT-tagged PRRSV screening system can omit the step of serial dilution for viral tittering and save more than 2 days to evaluate the inhibition effect of antiviral reagents, which greatly improves the efficiency of screening. Collectively, these data suggest that the split-luciferase HiBiT-tagged PRRSV generated can be easily applied for antiviral reagents screening.

### Set a novel luciferase-based virus neutralization assay with greatly improved efficiency.

**FIG 4:**
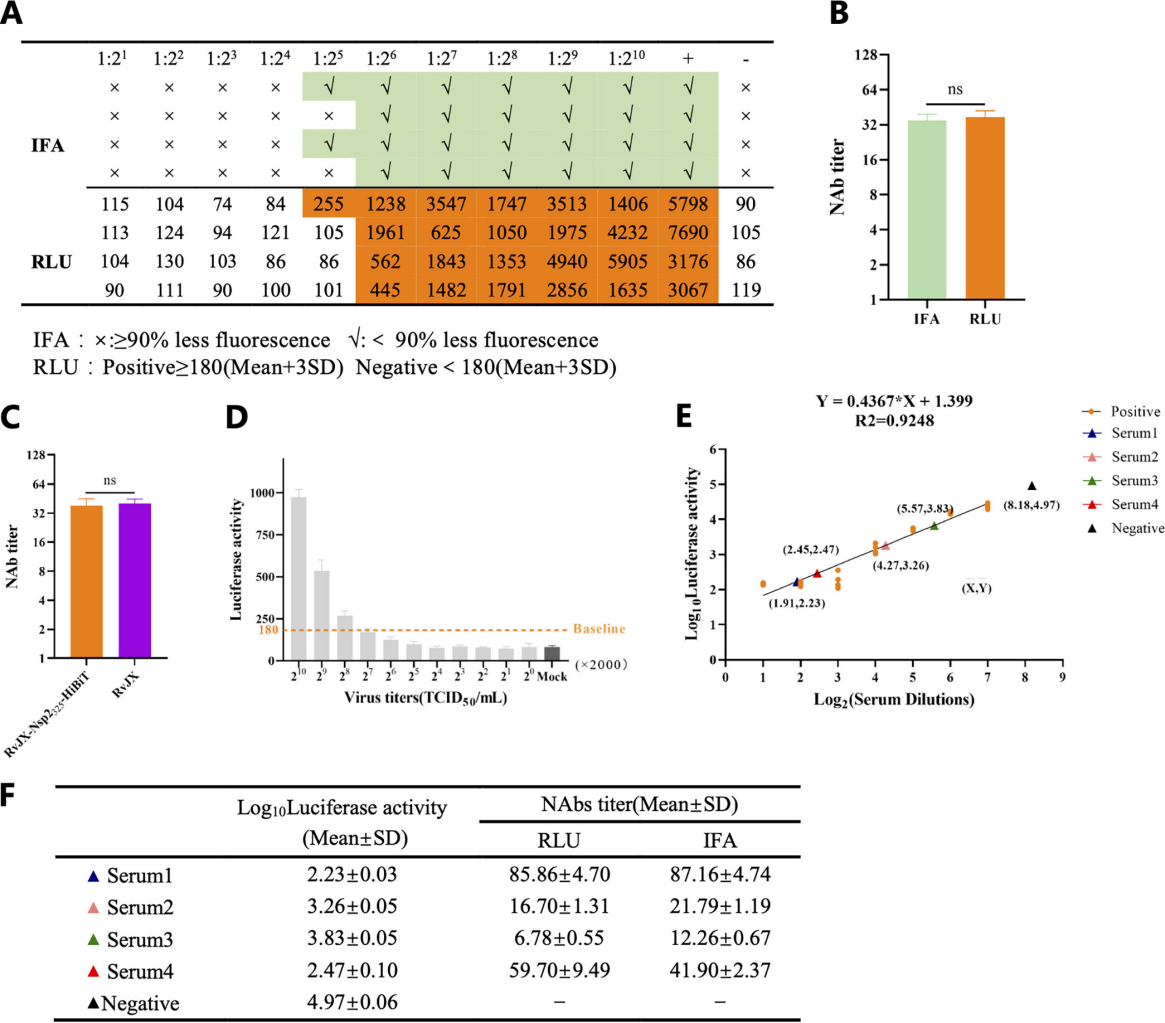
Application of the HiBiT-tagged PRRSV for neutralizing assay. (A, B) The neutralization titer of standard positive serum was determined by IFA and relative luminescence units (RLU), and three independent repeats were carried out. The RLU value of 180 was set as the cutoff to differentiate PRRSV positive or negative, which was calculated from the mean value of 20 mock wells treated with PRRSV negative sera. (C) Comparison of the neutralizing activity of standard positive serum against RvJX-Nsp2_325_-HiBiT and its parental virus RvJXwn. (D) Determining the lowest titer of RvJX-Nsp2_325_-HiBiT that the standard positive serum cannot completely neutralize. The value of 180 (mean ± 3 SD) was set as the fluorescent baseline to differentiate PRRSV as positive or negative. (E) Simple linear regression based on the intensity of luciferase activity at different dilutions of standard positive serum. Neutralizing antibody (NAb) titers of four PRRSV-positive sera were tested by luciferase-based assay and calculated according to the standard curve. (F) Compare the NAb titers tested by the novel luciferase-based assay and the conventional one based on IFA. ns, no significant difference.

Done conventionally, determination of PRRSV neutralizing antibody titer requires cumbersome dilution and time-consuming IFA, so only a small number of samples can be tested each time. Thus, it is urgent to develop a rapid and high-throughput assay to test the neutralizing antibody. To improve the test efficiency by using the HiBiT-tagged PRRSV, the luciferase activity was first confirmed to be able to replace IFA to determine the infection in the conventional neutralization assay. As shown in Fig. 4A and B, the serum neutralization titers calculated by IFA and relative luminescence units (RLU) were similar. However, only a few hours for IFA can be saved by this replacement, and it cannot eliminate the step of gradient dilution. A more efficient method is needed. The neutralization activity of the standard positive serum against the recombinant virus RvJX-Nsp2_325_-HiBiT and its parental virus RvJXwn was confirmed to be similar, shown in Fig. 4C. Then the lowest titer of RvJX-Nsp2_325_-HiBiT that the standard positive serum cannot completely neutralize (Fig. 4D) was determined as 2^8^ × 100 TCID_50_/50 μL, which was further used to react with the 2-fold gradient diluted standard positive serum and infected the MARC-145 cells. At 24 h postinfection (hpi), the luciferase activity value of virus neutralized with serum at different dilutions was detected, and the results show that the luciferase signal of standard serum gradually increases with the gradient dilution, and a simple linear regression can be fitted with *R*^2^ = 0.9248 (Fig. 4E). To confirm the accuracy of this method, four PRRSV-positive serum samples and one negative control serum from a specific-pathogen-free (SPFs) pig were submitted for determining their neutralizing antibody (NAb) titers by this assay and compared with the conventional one. The data of these two methods were very close, with a consistent trend (Fig. 4F). Considering there is no gradient dilution process in this method, it can easily be used for high-throughput testing of the PRRSV NAbs.

### The HiBiT-tagged PRRSV can still replicate *in vivo* and cause disease.

**FIG 5:**
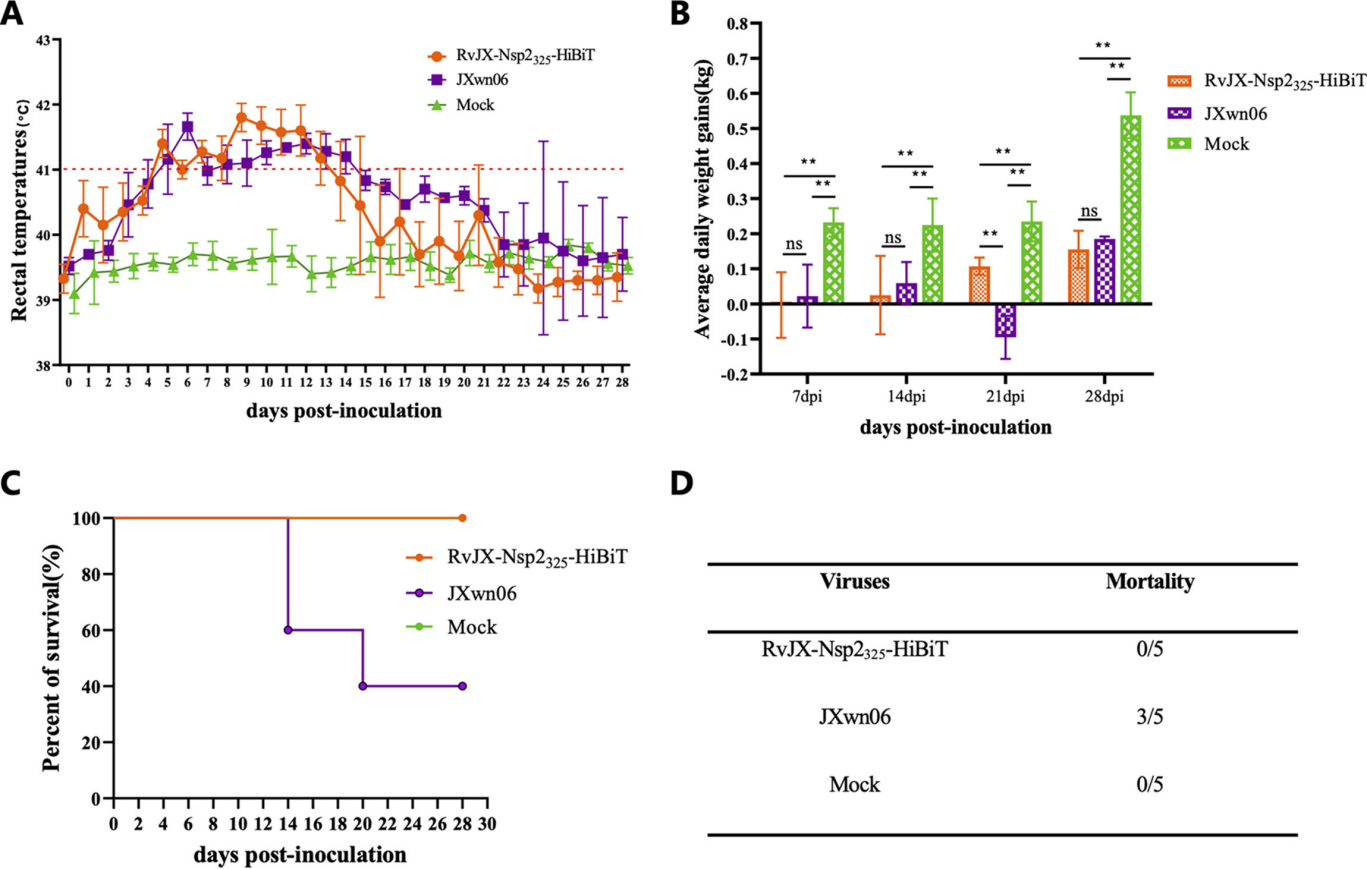
The clinical symptoms and mortality of inoculated pigs. (A) The rectal temperatures of pigs in the groups RvJX-Nsp2_325_-HiBiT, JXwn06, or mock were daily recorded. (B) The average daily weight gain (ADG) of inoculated pigs was calculated based on the weekly record of body weight. Asterisks indicate a significant difference in ADG between inoculation and mock groups. *, *P < *0.05; **, *P < *0.01. (C, D) The survival curves and mortality of each group were observed.

To further examine the influence of the HiBiT tag on the viral replication and virulence *in vivo*, the animal inoculation experiment was executed with 4-week-old pigs, and the growth characterization, as well as the pathogenicity of the recombinant virus RvJX-Nsp2_325_-HiBiT and its parental virus JXwn06, were investigated. The rectal temperature of inoculated pigs was recorded daily, and the data showed that both the RvJX-Nsp2_325_-HiBiT- and JXwn06-inoculated pigs developed similar symptoms of high fever (41°C above) around 5 to 13 dpi and had a peak temperature of nearly 42°C. Nevertheless, the rectal temperature of several pigs in the RvJX-Nsp2_325_-HiBiT-inoculated group rose 1 or 2 days earlier than that in the JXwn06-inoculated group, but they dropped down faster after 13 dpi (Fig. 5A). The average daily gain (ADG) of each group was calculated, and the ADG of both inoculated groups was remarkably lower than that of mock (Fig. 5B). During the first 2 weeks and the last week postinoculation, the ADG of pigs inoculated with RvJX-Nsp2_325_-HiBiT was even lower than that of the wild-type JXwn06 group with no significant difference, but during the third week postinoculation, the ADG of the RvJX-Nsp2_325_-HiBiT group was higher than that of JXwn06 group, in which the pigs even lost body weight. The mortalities of inoculated pigs were recorded and are shown in Fig. 5C and D. Three pigs in the JXwn06-infected group died within days 14 to 21 dpi, whereas all pigs inoculated with RvJX-Nsp2_325_-HiBiT survived during the experiment period.

**FIG 6:**
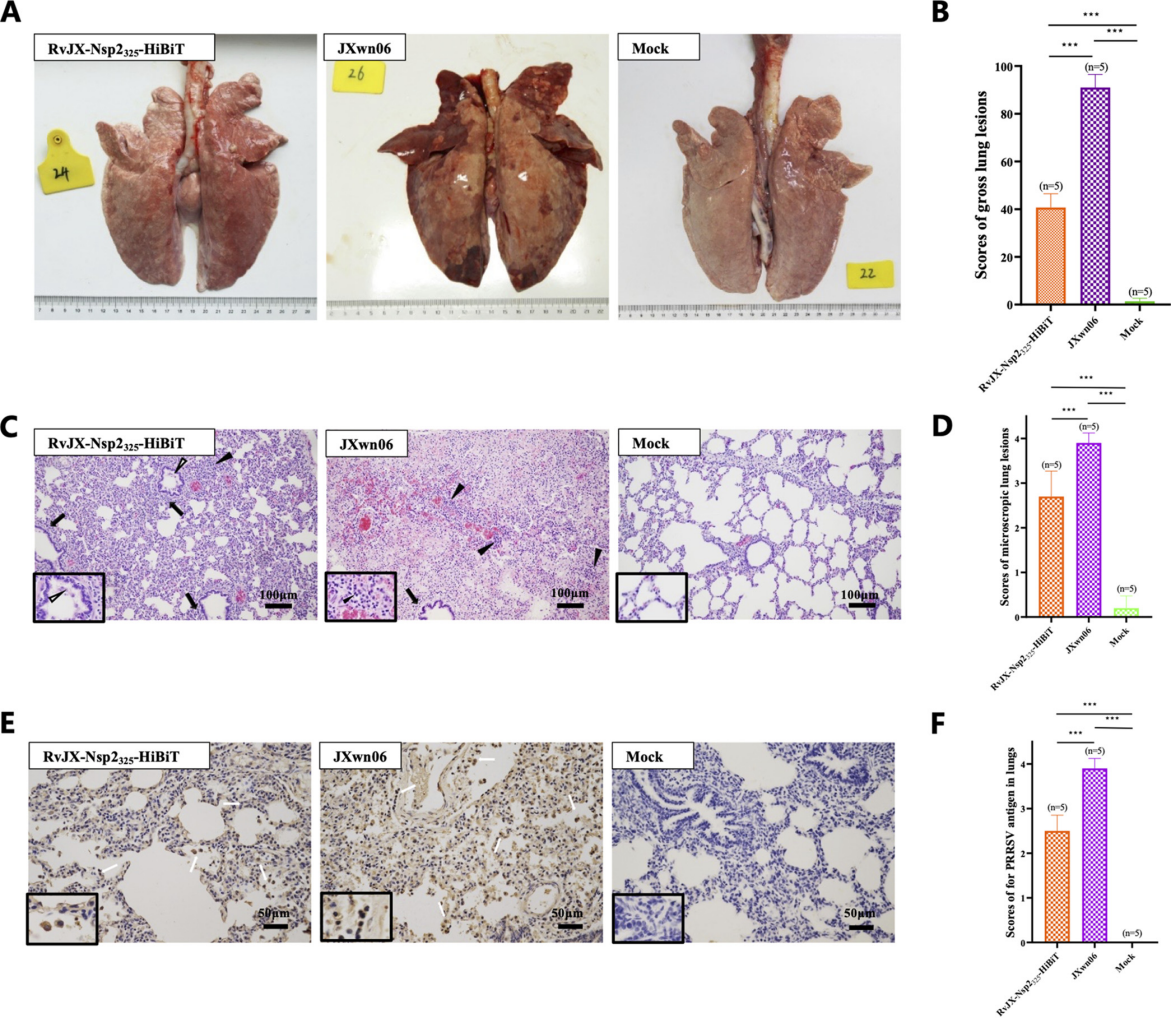
Lung lesions and immunohistochemical examination for PRRSV antigen in lungs. (A, B) Shown are the representative gross lung lesions and the gross lesion scores of each group. (C, D) Shown are the representative microscopic lung lesions and the microscopic lesion scores of each group. Solid arrows indicate thickening of the interlobular septal and infiltration of inflammatory cells around the bronchiole. Solid triangles indicate necrotic debris, inflammatory cells, and exfoliated epithelial cells infiltrate in the bronchiole. The open triangle represents hemorrhage or infiltration of inflammatory cells in alveolar spaces or alveolar septa. (E, F) Immunohistochemistry (IHC) was performed by using monoclonal antibodies specific for the N protein of PRRSV, and the scores based on numbers of positive cells in the lungs were evaluated. Representative views of immunohistochemistry examinations and mean scores of lungs of each group are shown. The macrophages are stained strongly dark brown as the PRRSV antigen. The open arrow represents positive signals in macrophages within or around the alveolus and bronchus.

Necropsies and gross lesion examinations were performed, once the pig died during the trials or after all survived pigs were euthanized at the end of trials (28 dpi). The typical HP-PRRS-related lung lesions including severe interstitial pneumonia with extensive and marked pulmonary edema, hemorrhage, and consolidation can be observed in both inoculated groups (Fig. 6A), but the mean score of gross lung lesions of RvJX-Nsp2_325_-HiBiT-inoculated pigs was significantly lower than that of JXwn06-inoculated pigs (Fig. 6B). The lung microscopic lesions indicated that two infection groups exhibited similar histopathological changes characterized by destroyed lung structure, thickened interlobular septal, lots of inflammatory cells, and necrotic debris-filled alveolar spaces and bronchioles. Generally, the level of microscopic lesions and virus distribution in the lungs of RvJX-Nsp2_325_-HiBiT-inoculated pigs was lower than that of JXwn06-infected pigs. Collectively, these results indicated that replacing the aa 325 to 335 regions of Nsp2 with the HiBiT gene does not impair viral infection *in vivo*, but it might attenuate its fatal virulence for pigs.

### Applicability of HiBiT recombinant PRRSV for monitoring viral dynamics *in vivo*.

The viremia of inoculated animals was examined by using a microtitration infectivity assay. The data showed that RvJX-Nsp2_325_-HiBiT can grow as well as its parental virus JXwn06 at the first week postinoculation, but viral titers of HiBiT-tagged virus were lower than that of JXwn06 during the second and third weeks postinoculation, indicating it was earlier to be cleared by the host, which corresponded to the changes of ADG and rectal temperatures during that period.

**FIG 7:**
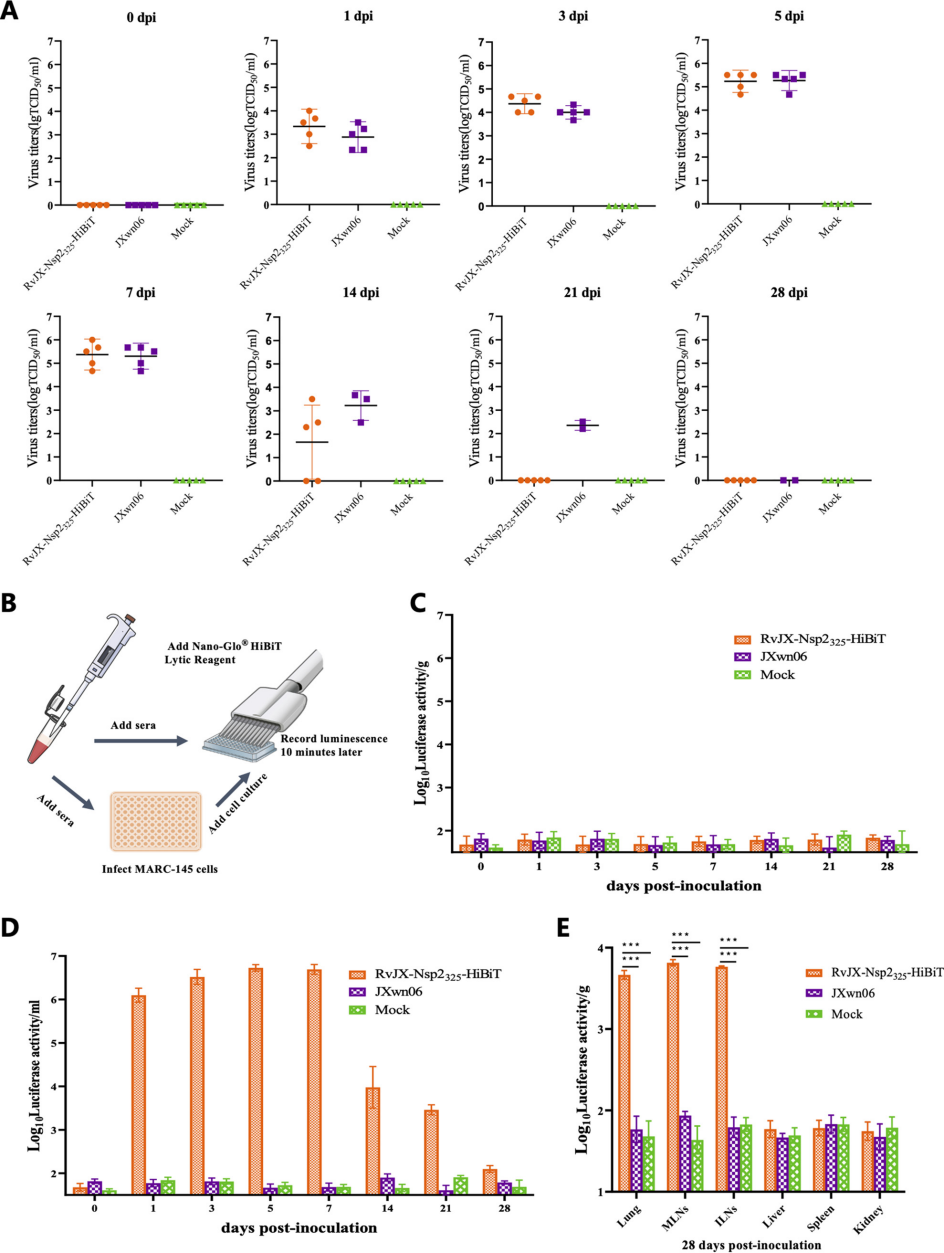
Viral dynamics of HiBiT-tagged PRRSV *in vivo*. (A) Viral loads of viremia of the pigs inoculated with the HiBiT-tagged PRRSV, parental virus, and mock. The infectious titers in the sera of each time point were determined by endpoint dilution assay and shown as TCID_50_/mL. (B) Diagram of two ways to detect luciferase signals of HiBiT-tagged PRRSV from serum samples. (C) Detecting the luciferase activity of serum sample by using Nano-Glo HiBiT lytic reagent directly. (D) The serum samples were first added to the MARC-145 cells for 36 h inoculation, and then the luciferase activity was detected. (E) The luciferase activity of tissue samples from inoculated pigs was directly determined by using Nano-Glo HiBiT lytic reagent. Asterisks indicate a significant difference between inoculation and mock groups, on the luciferase activity of different tissues. *, *P < *0.05; **, *P < *0.01; ***, *P < *0.001.

To evaluate the utility of the luciferase-tagged recombinant PRRSV for monitoring viral dynamics *in vivo*, the collected serum and tissue samples of the inoculated pigs were submitted for luciferase activity assay. Two strategies were designed in this study: one was to directly measure the luciferase activity of the serum samples, and another one was to inoculate MARC-145 cells with collected serum samples and test luciferase activity at 36 hpi (Fig. 7B). Unsurprisingly, only background fluorescent signals can be detected in all serum samples by direct test; however, the luciferase activities can be detected from the supernatant of MARC-145 inoculated with serum samples (Fig. 7C and D). Moreover, the luciferase activity of RvJX-Nsp2_325_-HiBiT can be also detected in the pig tissues, such as lungs, mandibular lymph nodes (MLNs), and inguinal lymph nodes (ILNs) (Fig. 7E), suggesting that the HiBiT gene was maintained in the recombinant virus while it infected the host, and viral dynamics *in vivo* could be successfully evaluated by using a luciferase assay. Taken together, these results indicated that this luciferase-tagged virus can be extensively applied for monitoring viral dynamics in the serum and tissue of the inoculated pigs.

## DISCUSSION

Currently, the vaccine is widely used on pig farms for PRRS prevention and control; however, the genetic variation of PRRSV limits the vaccine efficiency of cross-protection against heterological field strains. Meanwhile, the risk of reversion to virulence from its modified live vaccine raises some concerns about its utilization, so scientists and veterinarians are trying to find out some new strategies to control this disease, especially caused by variated strains. Some reagents that are able to nonspecifically inhibit the PRRSV proliferation, such as the macrolide antibiotic tylvalosin tartrate, have been practically used for treating herds with PRRS outbreak (32). However, there is still no real antiviral drug available for PRRSV. Therefore, some high-throughput approaches for screening and evaluating antiviral reagents are greatly needed for drug discovery.

Reporter genes, such as fluorescent and luciferase tags, have been used to visualize the intracellular distribution of viral protein, monitor infection events, or quantitatively detect viral protein expression or virus proliferation. The key point of successfully constructing the recombinant virus with reporter genes is to choose a suitable loci for reporter gene insertion or replacing viral fragments. To increase the possibility of successfully rescuing the recombinant virus and screening out the virus with the highest expression level of the reporter gene, five loci based on two construct strategies were set to insert the HiBiT fragment into the PRRSV genome. Among the nonstructural proteins of PRRSV, Nsp2 is viable and highly heterogeneous, suggesting that this region is tolerant for inserting foreign genes (33–35). However, the stability of HiBiT inserted in the Nsp2 coding region was initially concerned at the beginning of this project, so the loci between ORF7 and 3′-UTR were also selected to insert reporter genes as a separate transcription unit derived by an extra transcription regulatory sequence (TRS). At last, five recombinant viruses were successfully rescued. Generally, these five strains can grow well in the cells, just as their parental virus RvJXwn and the titers of RvJX-Nsp2_325_-HiBiT and RvJX-ORF7-HiBiT were as high as that of RvJXwn. However, the luciferase activity of RvJX-ORF7-HiBiT was a little lower than the other four strains, which is speculated to relate with the lower transcription level of this additional ORF or some individuals of the quasispecies lost this nonessential ORF during proliferation, the mechanism is unclear but worthy of study in the future. As a result, the recombinant strain RvJX-Nsp2_325_-HiBiT was selected as the representative one for the rest of the study.

Stability is another prerequisite for a good viral reporter system. As mentioned above, a recombinant PRRSV carrying GFP fused with the Nsp2 protein lost the green fluorescent protein after only seven serial passages in cells, due to the accumulation of mutants or the deletions. The insertion of a large fragment of the reporter gene into a viral genome presents the risk of impairment of viral growth or genetic stability. The split-luciferase NanoBiT provides a good solution, which only needs to insert an 11-amino-acid coding region into the viral genome. The serial passaging data of RvJX-Nsp2_325_-HiBiT indicated that the HiBiT gene is genetically stable *in vitro*, and this has been further confirmed in the inoculation experiment, as the environment *in vivo* can provide more complicated, restricted selection pressure for the recombinant virus, including the interaction with the host immune system. Interestingly, the original sequence of aa 325 to 335 region in Nsp2 is conserved among highly pathogenic PRRSV (HP-PRRSV) strains such as JXA1 and HuN4, and there were no deletions nor mutations found in this region while JXwn06 was serial passaged to P80 in MARC-145 cells. The data demonstrated that the NanoBiT luciferase system is applicable to PRRSV, as the HiBiT recombinants are genetically stable and exhibit similar replication characteristics with its parental backbone virus both *in vitro* and *in vivo*.

It is noticeable that the luciferase signal of recombinant PRRSV was hard to detect in the culture supernatant before cell fragmentation or directly from the serum samples of inoculated pigs. They might be reasonable, as the reporter protein is fusion expressed with viral nonstructural protein; however, PRRSV Nsp2 has been identified as a virion-associated structural protein and exits in or on viral particles in the previous study (36, 37), so we are wondering if this 11-amino-acid HiBiT can be assembled into the viral particle or not, and the undetectable issue is because HiBiT cannot be exposed to LgBiT or the amount of virion in the supernatant or sera is not enough. This might be a good clue for studying the function of Nsp2 in virion assemble in the future. In addition, the structural proteins of PRRSV are highly conserved in gene length and cannot easily tolerate the insertion or replacement of foreign genes. Previously, a recombinant PRRSV possessing a hemagglutinin (HA) tag fused with its ORF7 was reported; however, it lost the foreign HA epitope only after two round passages in cells (36). Therefore, more strategies need to apply if we want to determine the suitable sites for inserting luciferase reporters into PRRSV virions.

To evaluate the feasibility and efficiency of this HiBiT recombinant PRRSV as an unbiased high-throughput screening platform for anti-PRRSV reagents, a proof-of-concept study was performed by using four potential antiviral reagents: remdesivir, sofosbuvir, NHC, and ribavirin. Both luciferase activities and viral titers of inoculated cells were determined at 36 h postinfection, with the treatment of these reagents, and the changing trend of luciferase activity was consistent with viral titer in all treated groups, which indicated that remdesivir, NHC, and ribavirin show the various level of anti-PRRSV activity *in vitro*, but the sofosbuvir did not. Taking the advantage of reduced time-consumption and increased throughput to monitor the HiBiT-tagged PRRSV proliferation, this system will be a convenient tool for screening antiviral reagents.

For many viruses, the NAbs are a key part of humoral immunity against viral infection, but their role in controlling PRRSV is controversial. In a primary infection, PRRSV-specific NAbs are usually detected after the viremia is already resolved. On the other hand, the passive transferred homologous NAbs were shown to prevent reproductive disease and vertical transmission to offspring (38), and high titers of broadly neutralizing activity were reported to provide cross-protection against heterologous strains (39, 40). Generally, many aspects of the mechanism of PRRSV neutralization are still unknown. Highly efficient NAbs screening and evaluating tools are needed to explore them. In the conventional PRRSV virus neutralization test (VNT), the gradian dilution of testing serum samples and IFA observed under microscope greatly limits its throughput and testing efficiency. In this study, a novel assay was successfully generated based on the luciferase activities and the standard curve created by using a standard positive serum with a high level of PRRSV NAbs. Several serum samples with various PRRSV NAbs titers were used to prove the consistency of these two assays. As a proof-of-principle test, it has demonstrated the robustness of the assay; however, follow-up studies on larger numbers of samples from clinical pigs are still required to fully characterize this assay.

In previous studies, most recombinant PRRSVs with reporter genes were only studied *in vitro*; to evaluate the viral proliferation, virulence, and the validity and stability of HiBiT expression *in vivo*, the animal inoculation test was carried out in this study. The results indicate that RvJX-Nsp2_325_-HiBiT-inoculated pigs showed no mortality, lighter clinical signs, shorter duration of viremia, and high fever compared with the JXwn06-inoculated group, suggesting that the replacement of an 11-amino-acid subunit in Nsp2 (aa 325 to 335) impaired PRRSV fatal virulence in pigs. Interestedly, the viral titer of both RvJX-Nsp2_325_-HiBiT and JXwn06 were similar during the first week postinoculation, indicating that RvJX-Nsp2_325_-HiBiT retained its proliferation well *in vivo*, showing consistency in that it displayed indistinguishable growth kinetics from the parental virus RvJXwn in both MARC-145 cells and PAMs. However, the viremia of RvJX-Nsp2_325_-HiBiT can be cleared more quickly by the host after the second week postinoculation, which might be due to inducing a different immune response compared with its parental strain, JXwn06. Fortunately, the luciferase activity can be directly detected from tissues or after inoculating MARC-145 cells, indicating that the HiBiT gene retains its genetic stability in the recombinant PRRSV, even during infection *in vivo*. Just as in early stages of inoculation *in vitro*, no luciferase signal can be detected from the supernatant before freeze-thawing. We prefer that the amount of HiBiT-tagged Nsp2 “leaked” from infected target cells into the serum is too low to detect, compared with a higher concentration in tissue homogenate. Even though the luciferase activity cannot be directly detected from serum, the titer of viremia can still be obtained more easily through TCID_50_ assay by detecting the increased luciferase activity to indicate the PRRSV infection compared with observing the IFA signal or CPE in the traditional assay. Summarily, the HiBiT-tagged virus is still a convenient tool for investigating viremia dynamics and tissue tropism in infected animals.

To conclude, serial recombinant PRRSVs harboring a split-luciferase gene HiBiT were constructed and rescued in this study, and the feasibility, genetic stability, and efficiency for viral quantification both *in vitro* and *in vivo* were characterized by using one represent strain. The results demonstrated its advantages in screening antiviral reagents and NAbs. Furthermore, it will greatly facilitate the mechanistic study of PRRSV replication and pathogenesis in the future.

## MATERIALS AND METHODS

### Ethical statements.

The animal experiments in this study were carried out according to the Chinese Regulations of Laboratory Animals: The Guidelines for the Care of Laboratory Animals (Ministry of Science and Technology of the People’s Republic of China) and Laboratory Animal Requirements of Environment and Housing Facilities (National Laboratory Animal Standardization Technical Committee). All protocols for primary PAMs preparation and animal inoculation were approved by the Laboratory Animal Ethical Committee of China Agricultural University under approval No. AW81801202-2-1.

### Cells, viruses, and plasmids.

The MA104-derived monkey kidney cell line MARC-145 was cultured in Dulbecco’s modified Eagle’s medium (DMEM) (Gibco) with 10% fetal bovine serum (FBS) (Gibco) and penicillin (50 U/mL) and streptomycin (50 μg/mL) at 37°C under a humid 5% CO_2_ atmosphere. PAMs, the target cells of PRRSV, prepared from 1-month-old SPF pigs were maintained in RPMI 1640 (Gibco) medium, containing 10% FBS and penicillin (50 U/mL) and streptomycin (50 μg/mL) as previously described (41). The full-length infectious cDNA clone plasmids (pWSK-JXwn) of HP-PRRSV JXwn06 (GenBank accession number EF641008) and the rescued viruses (RvJXwn) were used in this study (33).

### Construction of recombinant full-length cDNA clones.

To construct a luciferase-tagged recombinant virus, two strategies were designed to insert the high-affinity NanoBiT tag (VSGWRLFKKIS, HiBiT) into the PRRSV genome as the fusion expressed protein or an individual ORF. According to previous reports on PRRSV genomic modification and our predictive analysis, four loci in the Nsp2 coding region at aa 12 to 22, aa 325 to 335, aa 338 to 348, and aa 419 to 429 were initially selected to replace the nucleotide with NanoBiT tag by overlapping fusion PCR (Fig. 1A) (34). In another strategy, the HiBiT luciferase tag, with an additional TRS2 to drive its transcription, is inserted between the ORF7 and the 3′-UTR of the viral genome through introducing two unique restriction sites AflII and MluI at its flanking region (Fig. 1B) (23). The sequences of the primers used for construction are listed in Table 1.

**TABLE 1:** Primers used in this study

Primer	Sequence (5′ → 3′)	Position
pWSK-JXwn-Nsp2_12_-HiBiT etc. construction		
JAF	GTATTTAAATACCGTCATGACGTAT	1 to 25
JAR	GCTTCGAAATTTGCCTGATCTTTAG	4801 to 4825
HiBiT_12_-F	GTGAGCGGCTGGCGGCTGTTCAAGAAGATTAGCGCTCATGAAACCCGGCAGGC ^*a*^	1372 to 1424
HiBiT_12_-R	GCTAATCTTCTTGAACAGCCGCCAGCCGCTCACACCAGAGCGTGGTTTCCTTG	1352 to 1404
HiBiT_325_-F	GTGAGCGGCTGGCGGCTGTTCAAGAAGATTAGCAATTGCTATTACCCTGCACA	2311 to 2363
HiBiT_325_-R	GCTAATCTTCTTGAACAGCCGCCAGCCGCTCACCTTGCCCAAAGGCTCTTGAG	2291 to 2343
HiBiT_338_-F	GTGAGCGGCTGGCGGCTGTTCAAGAAGATTAGCCGTGAGAGGTTAAATTCCGT	2350 to 2402
HiBiT_338_-R	GCTAATCTTCTTGAACAGCCGCCAGCCGCTCACGCAATTGGACAGTGAGAAGG	2330 to 2382
HiBiT_419_-F	GTGAGCGGCTGGCGGCTGTTCAAGAAGATTAGCCCACCCCCTCCACCAAGAGT	2573 to 2625
HiBiT_419_-R	GCTAATCTTCTTGAACAGCCGCCAGCCGCTCACTTTTAAATCGACCTGCTCAG	2593 to 2645
pWSK-ORF7-HiBiT construction		
ORF7-F	CGCCACAGCATCACCCTCAGCATGACTTAAGTGGGACGCGTTGGGCTGGCATTCTT	15147 to 15201
ORF7-R	ACGCGTCCCACTTAAGTCATGCTGAGGGTGATGCTGTGGCGCGGATCAGACGCACA	15132 to 15186
AflII-TRS2-HiBiT-MluI-F	TTAAG**TTGAACCAACTTTAGGCCTGAATTGAA**ATGGTGAGCGGCTGGCGGCTGTTCAAGAAGATTAGGTGA ^*b*^	15172 to 15248
AflII-TRS2-HiBiT-MluI-R	CGCGTCAGCTAATCTTCTTGAACAGCCGCCAGCCGCTCACCATTT**CAATTCAGGCCTAAAGTTGGTTCAA**C	15172 to 15248

a The sequences of HiBiT genes are underlined.

b The sequences of TRS2 are indicated with bold type.

### Recovery of viruses.

To rescue the recombinant viruses, the full-length plasmids were respectively transfected into MARC-145 cells by using Lipofectamine LTX reagent (Thermo Fisher), according to the manufacturer’s instructions. After 4 to 5 days of incubation at 37°C under a humid 5% CO_2_ atmosphere, the cells and supernatant were collected and freeze-thawed once; then the supernatant was harvested and serially passaged in MARC-145 cells for three passages. The rescued viruses were examined by indirect immunofluorescence assay (IFA) using the PRRSV anti-N monoclonal antibody (McAb) SDOW17 (Rural Technologies). To further verify the correctness of the HiBiT gene, the RNAs of third-passage recombinant viruses were extracted and subjected to reverse transcription (RT)-PCR, followed by sequencing. The rescued viruses were named RvJX-Nsp2_12_-HiBiT, RvJX-Nsp2_325_-HiBiT, RvJX-Nsp2_338_-HiBiT, RvJX-Nsp2_419_-HiBiT, and RvJX-ORF7-HiBiT, respectively.

### Virus replication kinetics in MARC-145 cells and primary PAMs.

MARC-145 cells and primary PAMs were infected with recombinant viruses at the same multiplicity of infection (MOI) of 0.1. After 1.5 h of incubation at 37°C, unbound viruses were washed off with PBS, and then the cell cultures were supplemented with maintenance medium (DMEM for MARC-145 or RPMI 1640 for PAMs) containing 2% FBS. The viruses were cultured until 72 h postinoculation (hpi), and at 12-h intervals, the inoculated cells with their supernatant were collected for titration on MARC-145 cells by using the endpoint dilution assay, recorded as 50% tissue culture infective dose per mL (TCID_50_/mL), according to the Reed-Muench method. The kinetics were analyzed based on the data from three independently repeated experiments.

### Luciferase assay.

The inoculated cells were subjected to freeze-thaw to release the HiBiT-tagged viral protein, and then the luciferase activity of the supernatant was measured by using a Nano-Glo HiBiT lytic detection system (Promega) according to the manufacturer’s instructions. Briefly, the Nano-Glo HiBiT lytic reagent is reconstituted by adding LgBiT protein (1:100) and substrate (1:50) to a detergent-containing buffer and mixed with collected cell supernatant. The luminescence generated is detected by the luminometer after a 10-min reaction.

### Stability of HiBiT gene in the recombinant virus during passaging in cells.

To test the stability of recombinant virus with HiBiT tag in cells, the recombinant virus RvJX-Nsp2_325_-HiBiT was serially passaged in MARC-145 cells to the eighth passages. The luciferase activity and virus titer of each passage were tested as above, and the HiBiT gene, together with its flanking sequences, was applied by RT-PCR and submitted for sequencing.

### Cell viability assay.

The cytotoxicity of remdesivir, sofosbuvir, NHC, and ribavirin was assessed by 3-(4,5-dimethyl-2-thiazolyl)-2,5-diphenyl-2H-tetrazolium bromide (MTT). Briefly, MARC-145 cells were treated with each reagent at a range of concentrations (6.25 to 200 μM) and incubated for 48 h at 37°C. Dimethyl sulfoxide (DMSO) was set as the negative control. Cell viability was tested using an MTT cell proliferation and cytotoxic assay kit (Solarbio, Beijing, China) following the manufacturer’s instructions. The 50% cytotoxic concentration (CC_50_) was calculated by using the software GraphPad Prism 8.0.

### Applicability of HiBiT recombinant PRRSV to screen antiviral reagents.

The efficiency of the HiBiT-tagged PRRSV for screening antiviral reagents was investigated. The confluent MARC-145 cell monolayers in 6-well plates were inoculated with HiBiT-tagged PRRSV (MOI = 0.1) in the medium with remdesivir, sofosbuvir, NHC, and ribavirin at a range of concentrations (10, 50, and 100 μM), respectively. After 1.5 h of incubation, the supernatant was removed, followed by three washes with PBS; then the medium with the same concentration of reagents as in the initial incubation was added. At 36 h postinfection, the luciferase activities and viral titers of inoculated viruses were determined by using Nano-Glo HiBiT lytic detection system and endpoint dilution assay, respectively. The average time consumed for each step was recorded for further comparison with the traditional method based on viral titration.

### Virus neutralization test.

Serum samples with a high level of neutralizing antibody against PRRSV were prepared by repeatedly inoculating the pigs with HP-PRRSV strain JXwn06 in our previous study (42). They were heat-inactivated at 56°C for 30 min, and then virus neutralization assays were conducted as previously described (43). Briefly, serum samples were serially diluted 2-fold with DMEM and then incubated with an equal volume of RvJX-Nsp2_325_-HiBiT or RvJXwn at the titer of 2 × 10^3^ TCID_50_/mL. After 1 h of incubation at 37°C, the mixture of virus and serum was transferred to a 96-well cell culture plate (100 μL/well) seeded with monolayered MARC-145 cells. After 1 h inoculation at 37°C, the cells were washed triple times with PBS and then added 100 μL 2% FBS DMEM to each well. At 24 hpi, PRRSV-positive wells were detected by IFA or Nano-Glo HiBiT lytic detection system recorded as RLU. The NAbs titer against RvJX-Nsp2_325_-HiBiT or RvJXwn were calculated using the Reed-Muench method.

### Set a luciferase-based virus neutralization assay.

One stocked PRRSV-specific pig serum with the NAbs titer of 1:2^5^ was set as the standard positive serum. The lowest amount of PRRSV that the serum cannot neutralize was first determined by reacting with the RvJX-Nsp2_325_-HiBiT, with 2-fold serially dilution from 2^10^ to 2^0^ TCID_50_/mL. The lowest titer of RvJX-Nsp2_325_-HiBiT that the standard positive serum cannot completely neutralize was further used for reacting with the 2-fold serially diluted standard positive serum. At 24 hpi, the neutralization effect was determined by testing the luciferase activity of each well, treated with serum at different concentrations. Then the standard curve of NAbs was built based on the value of luciferase activity and serum dilutions. And the *R*^2^ value was calculated to evaluate the quality of the standard curve. To confirm the accuracy of this method, the NAbs titers of four serum samples from PRRSV-infected pigs and one negative serum were parallelly detected by traditional virus neutralization test with serial dilution and IFA, as well as by RLU and the standard curve built with standard positive serum.

### Animal inoculation study of the recombinant virus.

To evaluate the pathogenicity of the recombinant virus, together with the availability and stability of the HiBiT tag *in vivo*, the animal inoculation study was further performed. Fifteen 4-week-old SPF pigs, obtained from the Beijing Center for SPF Swine Breeding and Management, were confirmed to be negative for PRRSV, pseudorabies virus (PRV), classic swine fever virus (CSFV), circovirus type 2 (PCV2), and Mycoplasma hyopneumoniae infections. The pigs were randomly divided into three groups (*n* = 5), and each group was separately raised in the isolated rooms in the animal facility of China Agricultural University (CAU). The pig in each group was intranasally inoculated with 2 × 10^6^ TCID_50_ virus (RvJX-Nsp2_325_-HiBiT or JXwn06) or 2 mL supernatant of MARC-145 cells as the negative control, respectively. The clinical symptoms, including respiratory disease scores and rectal temperature, were recorded daily, and the average daily weight gain (ADG) was calculated by weighting the pigs weekly.

Necropsy, gross and microscopic pathological evaluation, and immunohistochemistry (IHC) examinations were carried out once the pigs died during the experiment, and all of the surviving pigs were euthanized and necropsied on 28 dpi (44). Lung tissue samples were taken, fixed with 4% paraformaldehyde solution at room temperature for 48 h, and then processed by routine histopathological procedures. Each sample was examined in two sections: one section was stained with hematoxylin and eosin (H&E) for observing pathological lesions, and the other one was stained with the McAb SDOW17 at 1:1,000 dilutions for detecting PRRSV N antigen-positive cells. The scores of lung microscopic lesions were blindly evaluated from 0 to 4, which accounted for the distribution and severity of interstitial pneumonia. The IHC scores of PRRSV antigen were conducted through a range score of 0 to 4 for evaluating the numbers of PRRSV-positive cells as previously described (45).

### Viremia and viral load in tissues.

The blood samples were collected at 0, 1, 3, 5, 7, 14, 21, and 28 days postinoculation (dpi) to determine the viral loads in the sera of the infected animals by using a microtitration infectivity assay on MARC-145 cells. In parallel, the luciferase activity of the virus in the collected sera was measured by directly adding Nano-Glo HiBiT lytic reagent into serum samples or inoculating the MARC-145 cells with the sera for 36 h before testing the supernatant with Nano-Glo HiBiT lytic reagent. Furthermore, the tissue samples, including lung, MLNs, ILNs, liver, spleen, and kidney, were also collected after the pig died during the experiment or euthanized at the end of 28 dpi. The collected tissue samples were initially homogenized by a grinding mill with DMEM (with the ratio of 500 mg:1.5 mL), followed by freeze-thawing and centrifugation at 12,000 rpm/min for 15 min at 4°C. Then the supernatant was measured by luciferase assay as above.

### Statistical analysis.

All statistical analysis in this study was performed by GraphPad Prism (version 8.0) software. Statistical significance was evaluated by two-tailed unpaired Student’s *t* test, and the asterisks indicate the statistical significance. *p*<0.05 was considered statistically significant, and *p*<0.01 or *p*<0.001 means extremely significant. Error bars indicate means ± standard deviation (SD).
